# Automatic Characterization of the Physiological Condition of the Carotid Artery in 2D Ultrasound Image Sequences Using Spatiotemporal and Spatiospectral 2D Maps

**DOI:** 10.1155/2014/876267

**Published:** 2014-05-28

**Authors:** Hamed Hamid Muhammed, Jimmy C. Azar

**Affiliations:** ^1^School of Technology and Health (STH), Royal Institute of Technology (KTH), Alfred Nobels Alle 10, SE-141 52 Huddinge, Sweden; ^2^Centre for Image Analysis, Uppsala University, P.O. Box 337, 751 05 Uppsala, Sweden

## Abstract

A novel method for characterizing and visualizing the progression of waves along the walls of the carotid artery is presented. The new approach is noninvasive and able to simultaneously capture the spatial and the temporal propagation of wavy patterns along the walls of the carotid artery in a completely automated manner. Spatiotemporal and spatiospectral 2D maps describing these patterns (in both the spatial and the frequency domains, resp.) were generated and analyzed by visual inspection as well as automatic feature extraction and classification. Three categories of cases were considered: pathological elderly, healthy elderly, and healthy young cases. Automatic differentiation, between cases of these three categories, was achieved with a sensitivity of 97.1% and a specificity of 74.5%. Two features were proposed and computed to measure the homogeneity of the spatiospectral 2D map which presents the spectral characteristics of the carotid artery wall's wavy motion pattern which are related to the physical, mechanical (e.g., elasticity), and physiological properties and conditions along the artery. These results are promising and confirm the potential of the proposed method in providing useful information which can help in revealing the physiological condition of the cardiovascular system.

## 1. Introduction

Despite the promising fact that the consequences of cardiovascular diseases (CVD) have decreased considerably during the past two decades due to considering healthier lifestyles and using more efficient treatment regimens, these diseases are still accounting for at least 30% of global deaths, as can be, for example, found in Gebel [1]. In addition, these diseases are still the major cause of deaths in developed countries, as reported by Lloyd-Jones et al. [2] and Roger et al. [3]. Consequently, the costs associated with the diagnosis and treatment of CVD are already heavy and are still increasing every day. Therefore, the CVD risk stratification algorithms have recently gained increasing attention [4, 5]. In addition, it can be noticed that the majority of serious cardiovascular events occur in subjects or cases at low or intermediate risk [6, 7]. However, the population-based risk algorithms suffer from poor individual predictive ability. Therefore, there is an urgent need for more efficient screening tools to identify such vulnerable subjects or cases as early as possible to be able to benefit from considering drug treatment and changes towards healthier lifestyles (instead of considering surgical operations and other invasive treatment approaches). For example, a recent study showed that more than 50% of a group of randomly selected middle-aged individuals (that were apparently healthy) were actually suffering from subclinical atherosclerosis in the coronary or carotid arteries (without knowing about it). Therefore, screening of the general population, especially middle-aged and elderly subjects, for cardiovascular diseases is proven to be controversial [8].

The degradation or lack of elasticity in arterial vessel walls (i.e., their stiffness grade or severity) affects the blood pressure and flow and is directly or indirectly related to other cardiovascular consequences and serious events. Myocardial infarction, left ventricular hypertrophy, and other serious cardiovascular diseases and deficiencies (as well as other related serious events) can be caused by arterial wall stiffness (i.e., abnormal or lack of elasticity) [9]. For example, coronary heart disease and its consequences of increased arterial vessel wall stiffness can result in increased systolic blood pressure, increased pulse pressure, and increased mechanical load on the heart [10–12]. Another factor which is usually associated with the degradation of elasticity and consequently with increased arterial stiffness is ageing [13–15].

Arterial stiffness can be noninvasively evaluated by applanation tonometry which is a validated, reproducible, user-friendly, and low-cost method for this purpose [16, 17]. In addition, measuring the pulse wave velocity (PWV) at the carotid and femoral arteries is considered as the gold standard measure of arterial stiffness [18]. However, de Feyter [19] found a disadvantage in these approaches as they are mainly based on cross-sectional studies, and a more efficient approach, according to de Feyter [19], should also include longitudinal studies.

It has been shown in many research works (e.g., three examples can be found in [19–21]) that it is possible to identify the presence of coronary atherosclerosis and predict the risk of adverse coronary events, by using noninvasive imaging modalities for the evaluation of atherosclerotic disease in noncoronary peripheral vessels such as the carotid and the femoral arteries [22–24].

Recent research results indicate that degraded arterial vessel wall elasticity usually appears early, even before the appearance of any clinical symptoms or atherosclerotic plaques [25–27]. Therefore, most noninvasive approaches, which were proposed for the diagnosis of cardiovascular diseases, were based on evaluating and quantifying the elasticity or stiffness of arterial vessel walls.

However, the methods that were proposed for early and accurate diagnosis of cardiovascular diseases had over the years relied mainly upon invasive procedures. In 1990, Parker and Jones [28] introduced such a method based on the concept of wave intensity (WI) analysis. The wave intensity (WI) signal was defined as the product of *dP* × *dU*, where *dP* and *dU* represent the change in pressure and flow, respectively, inside a given blood vessel. Both parameters of the WI signal were measured invasively inside the arterial vessel, and this requirement limited the clinical use of the WI-analysis method.

A recently introduced technique by Larsson et al. [29], known as wave intensity wall analysis (WIWA), can be considered as an important development of the WI-analysis concept to make it noninvasive. The changes in pressure (*dP*/*dt*) and flow (*dU*/*dt*) were estimated noninvasively by approximating the strain rate of the arterial wall in the radial and the longitudinal directions, respectively, from ultrasound image sequences. However, all WI-analysis approaches and variants have several common disadvantages. At first, the basic concept of traditional WI-analysis is based on the simultaneous measurement of the change in blood pressure and the change in blood flow inside the carotid artery, which is a nontrivial task (Parker 2009 [30]). In addition, since the WI-analysis approach is defined as the product of two quantities that are highly contaminated with noise, the obtained results are doubly sensitive to noise (Parker [30]). The recent WIWA method which estimates these quantities from ultrasound image sequences suffers from inaccuracy due to the difficulty in estimating the longitudinal strain in general and also the difficulty in manually finding the right or most suitable region of the arterial vessel to perform the measurements and get usable and reliable results (Larsson et al. [29]). In addition, a serious issue with the WIWA concept is that the measured strain rates cannot be claimed to really or exactly correspond to changes in blood pressure and flow. In any case, WI-analysis remains limited in the sense that it cannot provide any insight into the (physical and physiological) conditions of arterial vessel walls in a local manner, and it is not concerned with visualizing the true wave progression along the vessel walls.

Therefore, the current study presented in this work suggests simplifying the analysis and limiting it to evaluating and quantifying the elasticity of the walls of the carotid artery by measuring its radial distension locally in small regions along it.

Many existing methods perform local radial-motion measurements of arterial stiffness by estimating the corresponding distensibility, compliance, elastic modulus, or stiffness index. However, none of these methods is free from limitations, and the validity and reproducibility of the performed measurements show large variation and nonrobustness [32, 33]. Recently, Azar and Muhammed [34] used a self-organizing neural network to automatically track the motion of the wall of the carotid artery in ultrasound image sequences. However, a serious disadvantage of that approach was the heavy computational load that was required by the used self-organizing neural network. In addition to that, the goal of this approach was to achieve an algorithm which results in a more efficient and practical WIWA approach. It aimed at automating the task of producing WIWA signatures of good quality, which could help achieve a more accurate and objective diagnosis. Therefore, this approach also suffered from the limitations of the WIWA technique.

Therefore, the aim of this work is to develop a method that can overcome the limitations of the existing ones when measuring deviations from normality. A previous attempt to achieve this goal was performed by Hamid Muhammed and Azar [31], where the best location on the carotid artery wall was automatically identified to be able to obtain a radial distension signal of accepted quality. Fourier analysis of the radial distension signals obtained for healthy and pathological cases could help differentiate between healthy and pathological cases. The achieved classification results were promising, showing significant differences between healthy young, healthy elderly, and pathological elderly cases. However, the dataset used for that study was limited and a larger dataset should be used for a more realistic and fair evaluation of that method. Therefore, the current work utilizes an extended dataset of that used in [31] to consider a population with higher variation of all three classes or categories of cases. The current paper describes a novel method for visualizing and characterizing the wave propagation along the whole vessel wall in an automated fashion. This approach can reveal and shed light on the wavy motion's mechanical properties along the carotid artery wall and aid in the accurate diagnosis of the cardiovascular system. The contributions of this paper are as follows.

1. An automated method for efficient and easy-to-understand visualization of wave propagation along arterial vessel walls is proposed. The approach captures both spatial and temporal variations in the radial direction of the vessel walls and reveals the characteristics of these wavy patterns.
2. The proposed method is shown to be able to aid in differentiating between healthy and pathological conditions, by visual inspection, in terms of the shape and the 2D pattern of the generated spatiotemporal map of the wave progression in each case. Such visual characteristics and differences between healthy and pathological cases can aid medical specialists in diagnosing the condition of the carotid artery and the cardiovascular system.
3. The Fourier transform is applied to the spatiotemporal 2D map to generate the corresponding spatiospectral 2D map. Visual inspection of the new map can reveal the spectral characteristics, which are related to the physical and mechanical properties, and consequently the physiological condition along the corresponding arterial vessel wall.
4. A novel approach is proposed to analyze the spatiospectral 2D maps in an automated manner to differentiate between healthy and pathological conditions of the arterial vessel walls.
5. Through extensive experiments and testing, the proposed noninvasive method is shown to be a robust, objective, and automated tool that can aid in achieving a reliable and accurate diagnosis of the physiological condition of the arterial vessel walls.

The results of analyzing the spatiotemporal and spatiospectral 2D maps are compared with the corresponding results obtained using the method proposed and implemented in [31]. Finally, the results of the two new approaches proposed in this work can be combined to achieve better results.

## 2. Materials and Methods

### 2.1. Three Datasets of Ultrasound Image Sequences

In this study, three datasets of ultrasound image sequences of the carotid artery were used. Each dataset consisted of healthy young cases, healthy elderly cases, and cases suffering from coronary artery disease (CAD). All CAD cases were verified by utilizing diagnostic coronary angiography. For this purpose, all coronary segments with all vessel diameters were assessed. Significant stenosis was diagnosed when there was a reduction of minimal lumen diameter by more than 50% as compared with the proximal reference.

The first dataset consisted of nine healthy young cases (31–45 years old) and eight healthy elderly cases (62–70 years old) where two of them were suffering from CAD. It was provided by the School of Technology and Health (STH), Royal Institute of Technology (KTH), in collaboration with Karolinska Institutet (KI), Stockholm, Sweden.

The second dataset consisted of 14 healthy elderly cases (56–69 years old, males and females) in addition to 20 pathological elderly cases (61–73 years old, males and females) suffering from CAD. It was provided by the Division of Cardiology, Rafik Hariri University Hospital, Beirut, Lebanon.

The third dataset was the largest and consisted of 21 healthy young cases (34–45 years old, males and females), 27 healthy elderly cases (49–67 years old, males and females), and 47 pathological elderly cases (48–77 years old, males and females) suffering from CAD. This dataset was provided by Ibn Al-Bitar Hospital for Cardiac Surgery, Ibn Al-Nafis Hospital for Cardiovascular Diseases, and the Iraqi Center for Heart Diseases, Baghdad, Iraq.

In these experimental studies, all participants, from all three countries, gave their informed consent to participate. These experiments were approved by the corresponding local ethics committees in Sweden, Lebanon, and Iraq.

### 2.2. Image Segmentation

Extracting the vessel wall in an ultrasound image sequence poses a considerable challenge mainly due to the low signal-to-noise ratio (SNR), unclear boundaries, and varying shape, location, and pixels' intensities across different frames.

**Figure 1:**
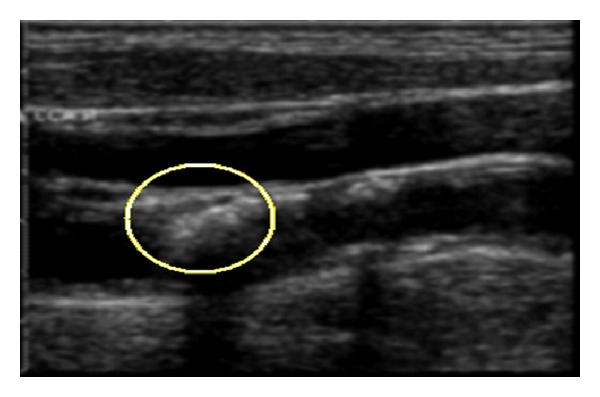
Carotid artery ultrasound image. The drawn ellipse marks a stenosis.

Therefore, the segmentation process should take advantage of both spatial and temporal information in the image sequence in order to segment the carotid artery wall in the images (i.e., the frames) accurately.  Figure 1 shows a typical image of a case with carotid artery stenosis (CAS), which occurs when some parts of this artery's walls become thicker and consequently get narrower (as marked by the ellipse in Figure 1).

**Figure 2:**
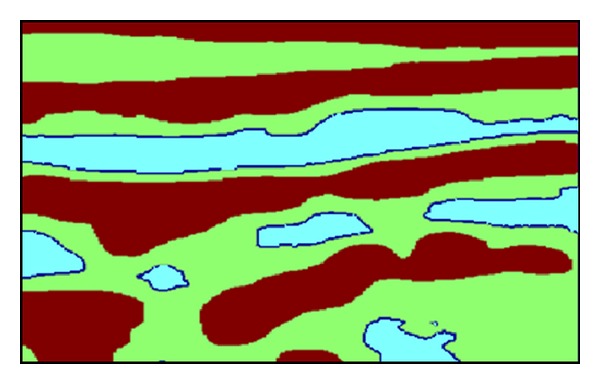
Segmentation by using *k*-means, with *k* = 3.

At first, each frame, which is a single still image, was clustered using a number (*N*) of certain spatial/textural features, where every pixel became an object or an element in an *N*-dimensional feature space. *N* was set to three in our case since the features chosen were the following: the mean, the standard deviation, and the entropy of a square neighborhood around each pixel. Based on these features, the *k*-means clustering algorithm was implemented to segment each frame into three classes, as shown in Figure 2.

In Figure 2, it is not easy to differentiate vessel wall boundaries from other boundaries, since the obtained sets of three features of these regions are very similar when considering only one frame. Even a human operator may not be able to distinguish or reveal any characteristic differences by processing only one such stationary image. However, when the images are set in a time sequence, the motion allows us to identify the vessel walls, since it is mainly the vessel walls that move while other boundaries and details remain relatively static (i.e., not moving). Therefore, to be able to achieve useful or acceptable results, both spatial and temporal information need to be integrated into the segmentation process. The additional feature, which was utilized for this purpose, was the standard deviation of the pixel values across the frames in the image sequence.

**Figure 3:**
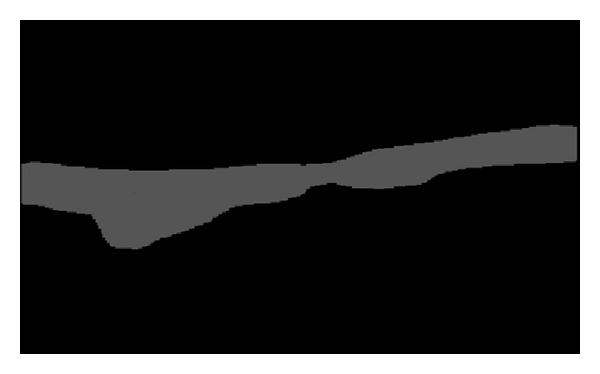
Extracted vessel wall.

At the end, this process ensures that the most prominent one, of the two vessel walls which appear in each frame, will be selected, that is, the one that is the most dynamic and moving more actively across all frames (by virtue of the standard deviation feature) and the most visible (by virtue of the *k*-means algorithm).  Figure 3 shows a segmented carotid artery wall using all four features mentioned previously (three spatial statistical features and one temporal statistical feature).

### 2.3. Automated Wave Tracking

**Figure 4:**
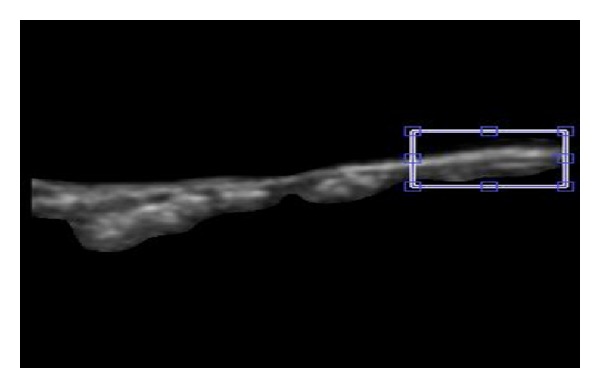
Manual cropping of a region of interest (ROI) marked by a rectangle.

The inspiration behind the idea of visualizing the wave propagation stems from the need for automated best-region search and selection so as to improve upon manual region selection in Larsson et al. [29] as well as Azar and Hamid Muhammed [34] in which the user is given the choice of cropping a region of interest (ROI) in the first frame, as shown in Figure 4.

**Figure 5:**
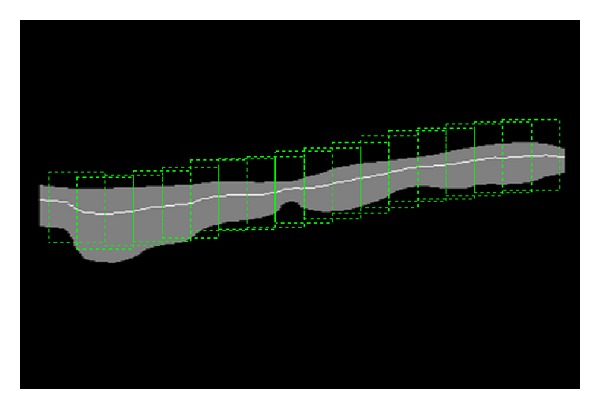
Regions overlapping midway are assigned along the longitudinal direction of the vessel wall.

Automated wave tracking is based on the systematic assignment and evaluation of different regions along the vessel wall, as described by Hamid Muhammed and Azar [31]. The method proceeds by, at first, effectively isolating the internal section of the vessel wall that is in contact with blood flow. Then, a series of regions of interest (ROIs) are assigned along the longitudinal direction of the vessel wall in a manner such that these ROIs overlap midway as shown in Figure 5.

### 2.4. Spatiotemporal Wave Representation

The basic concept is to obtain, from each region of interest (ROI) which is extracted as described in the previous section in Figure 5, a time dependent signal per ROI showing the radial variation at the corresponding ROI. However, in [31], this type of radial-variation signal was computed for only one ROI which is expected to give a usable signal of sufficient quality. If the vessel wall truly moves in a wavy pattern (as its inner layer is being affected and attracted by the blood flow), then it should be possible to track that wave as it moves along the vessel wall, that is, in the longitudinal direction. The concept of tracking the progression of this wavy motion will be now presented.

The systematic assignment of midway overlapping ROIs at different locations undertaken previously may be utilized exactly for this purpose. The idea is to simply show, in a three-dimensional (3D) plot, the variation in the radial direction (radial distension) as a function of both time (frame number) and space along the vessel wall (longitudinal location). Thus, the separate signals, each showing the variation of the radial thickness (e.g., the diameter) of the vessel as a function of time at a given ROI, are stacked beside each other (or concatenated behind each other) and presented in a 3D plot as a function of time and longitudinal position.

**Figure 6:**
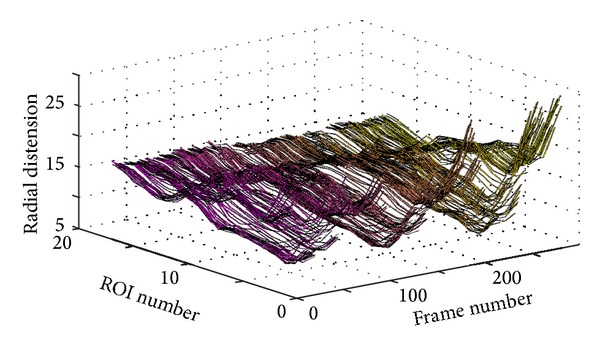
3D plot showing the wave propagation along the vessel wall at different ROIs for the healthy young case Y1.

**Figure 7:**
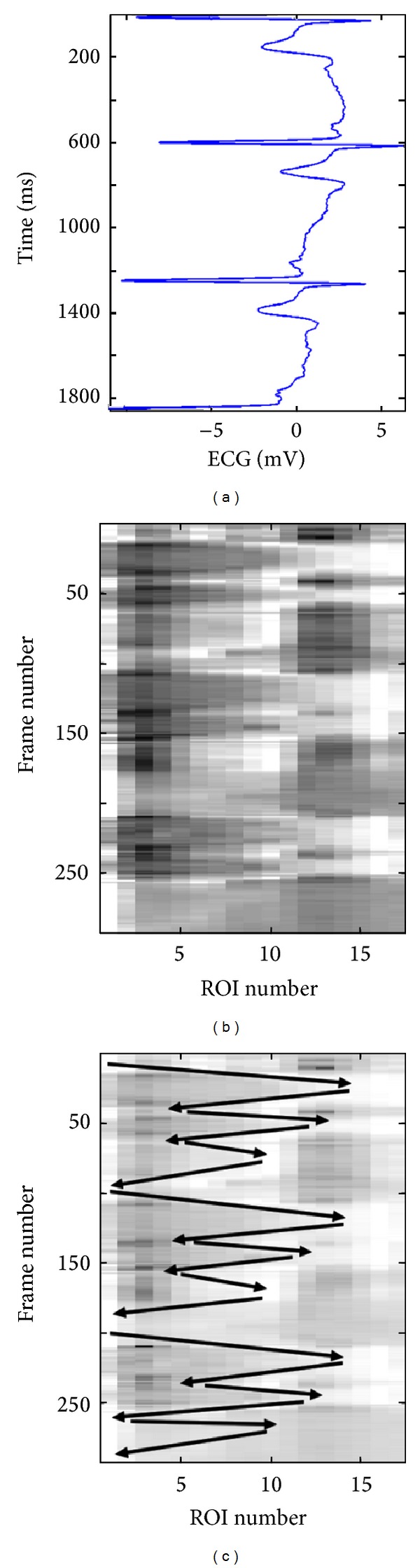
Spatiotemporal wave representation corresponding to the same healthy young case Y1 shown in Figure 6. The ECG signal is shown to the left (a) and the spatiotemporal 2D map is shown to the right (b). The longitudinal motion, across neighboring ROIs, is marked by black arrows on the spatiotemporal 2D map (c).

Figure 6 shows a 3D plot for a healthy young case (Y1). However, it is not easy to analyze this 3D plot by visual inspection. Perhaps the only observation that can be noticed, in this 3D plot, is the rapid radial variation across the frames. Therefore, another way to visualize the wavy pattern is proposed. The idea of the new approach is to show each 3D plot as a two-dimensional (2D) image or map using pseudo colors for the third dimension representing the radial vessel thickness at different locations (or positions) and time points. The echocardiography (ECG) signal is also utilized to be able to more exactly know when certain wavy patterns appear in the 2D map.  Figure 7(b) shows such a 2D map (in grey scale) corresponding to the same healthy young case presented in Figure 6.

Figure 7 gives evidence of how certain regions (ROIs) of the vessel wall move longitudinally in a wavy-like pattern across time. Comparing the 2D map in Figure 7(b) with the corresponding ECG signal in Figure 7(a) can help in characterizing this wavy pattern. It is, for example, easy to recognize three pairs of darker blobs on the left side of the image in Figure 7(b). Each such pair of darker blobs (followed by one light grey blob) is located within one ECG cycle (i.e., one heartbeat) as it can be noticed from Figure 7(a). The brighter boundaries of these blobs, which are almost connected, show the longitudinal motion of the carotid artery wall as a function of time. The black arrows in Figure 7(c) show how these boundaries and the corresponding longitudinal motion propagate with time in a zigzag manner, which is a result of the vessel wall boundary moving several times in the longitudinal direction to the right then backward to the left.

Comparison of this zigzag pattern with the corresponding ECG signal in Figure 7(a) can provide detailed information concerning the progression of the wave and the condition along the vessel wall locally at each ROI. It can, for instance, be observed that, within each ECG cycle, the vessel wall performs a longitudinal right-left motion three times. This motion pattern is repeated in almost an identical fashion during all three ECG cycles, as shown in Figure 7(c). Approximately, the same characteristic pattern appears in the spatiotemporal 2D maps of all healthy young cases examined in this study. This pattern is an indication that the vessel wall is nonrigid and flexible enough to exhibit a longitudinal right-left motion three times before completely relaxing to its initial position. Therefore, this characteristic pattern can be found in all healthy young cases, while it seems to be absent in all elderly and pathological (CAD) cases that were included and examined in this study.

**Figure 8:**
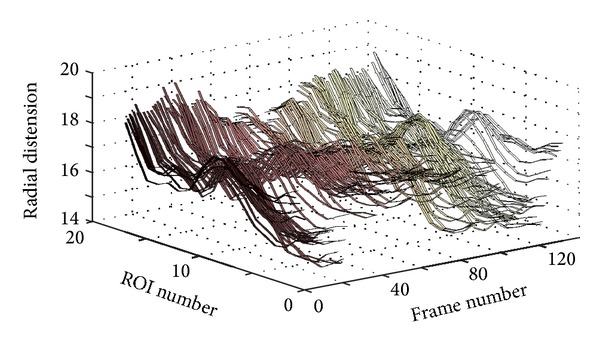
3D plot showing the wave propagation along the vessel wall for the pathological case CAD1 (coronary case 1).

**Figure 9:**
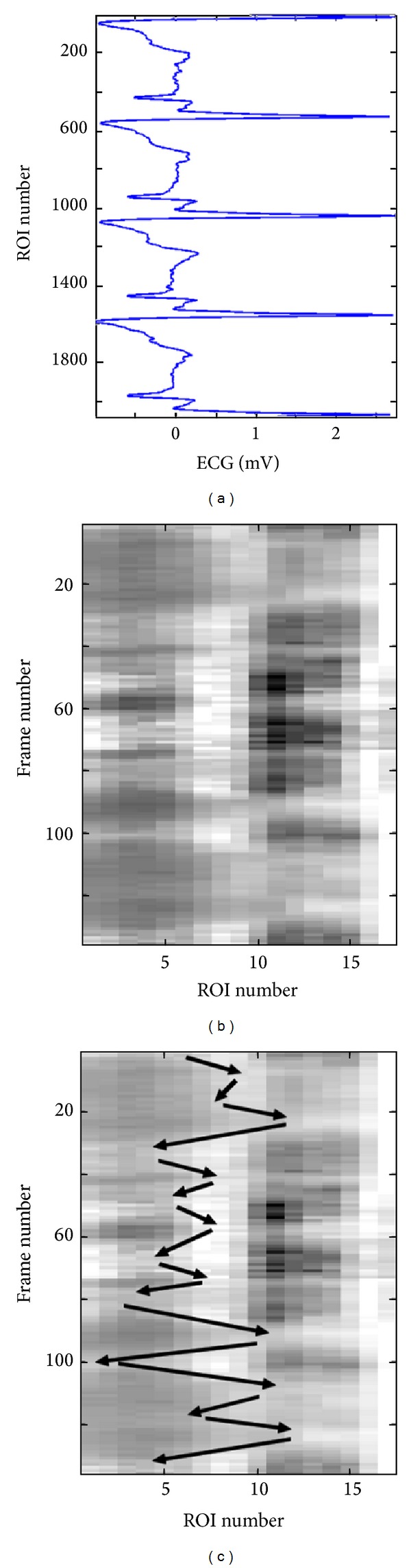
Spatiotemporal wave representation for the pathological case CAD1 (coronary case 1) in Figure 8. The ECG signal is shown to the left (a) and the spatiotemporal 2D map is shown to the right (b). The longitudinal motion, across neighboring ROIs, is marked by black arrows on the spatiotemporal 2D map (c).

Figure 8 shows a 3D plot for a pathological case (coronary case 1, CAD1). In this case too, as in the healthy young case presented in Figure 6, it seems that it is not easy to interpret this 3D plot, and the only observation that can be noticed is a rapid radial variation with time. Therefore, the corresponding 2D map is generated, as shown in Figure 9(b), to be used to provide a more easily interpreted spatiotemporal visualization approach. The corresponding ECG signal is also plotted in Figure 9(a) to be utilized to achieve a more accurate interpretation and to consequently obtain a more correct assessment or diagnosis of the physiological condition of the carotid artery. Since the plotted ECG signal shows four ECG cycles, it should be expected to recognize four repeated (almost identical) patterns in the corresponding 2D map to indicate that the current case is healthy. Note that approximately the same characteristic pattern should appear in all healthy cases. But, since that is not the case (as explained in Figure 9(c)), it is then possible to draw the conclusion that the case presented in Figure 9 does not seem to correspond to a healthy subject. The black arrows in Figure 9(c) indicate approximately how the longitudinal motion occurs. In the current case, the vessel wall performs a right-left motion twice within each ECG cycle. This motion seems to be irregular when comparing the obtained motion patterns within all four consecutive ECG cycles. Furthermore, during each ECG cycle, this double right-left pattern of coronary case CAD1 is much slower than the triple one obtained for the healthy young case Y1.

**Figure 10:**
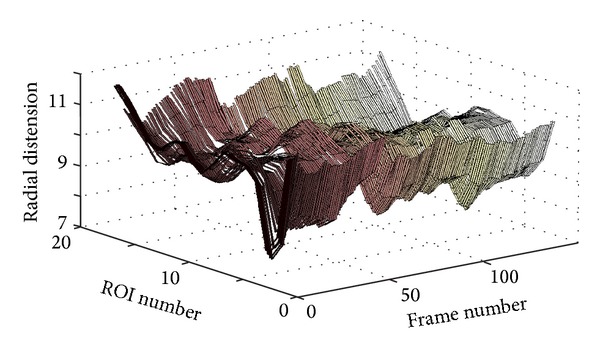
3D plot showing the wave propagation along the vessel wall for the pathological case CAD2 (coronary case 2).

**Figure 11:**
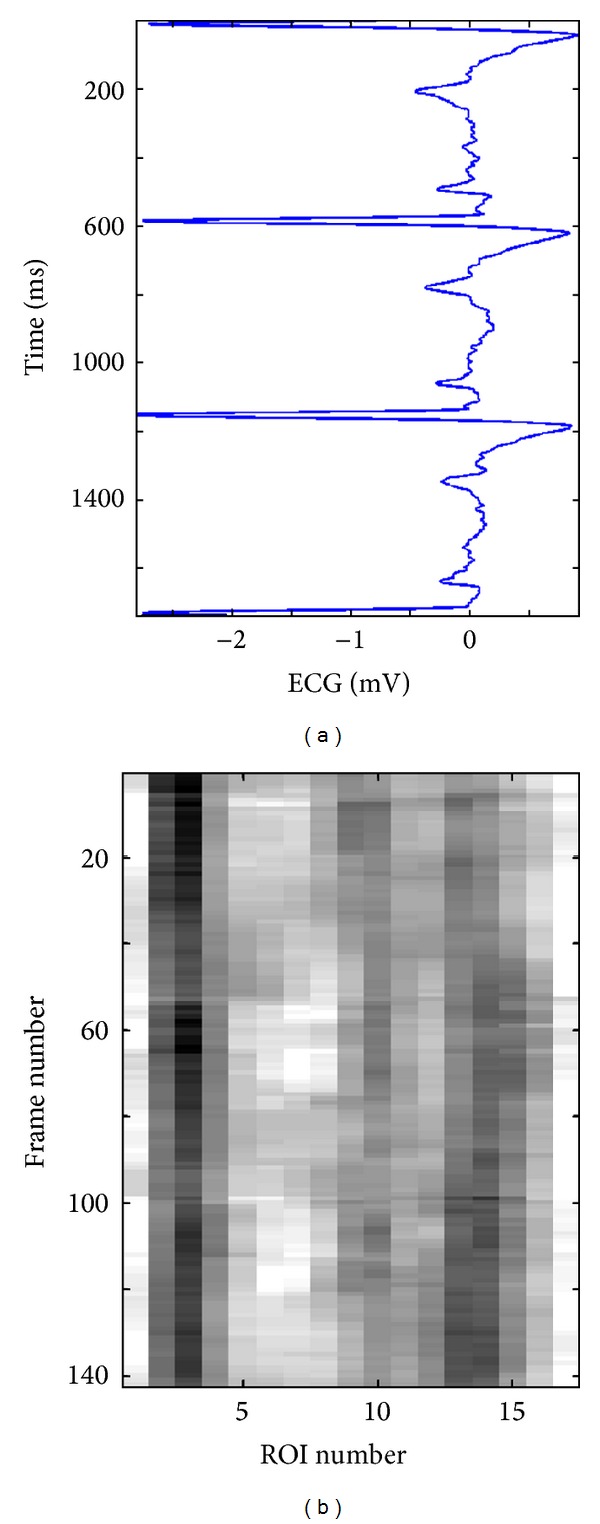
Spatiotemporal wave representation for the pathological case CAD2 (coronary case 2) in Figure 10. The ECG signal is shown to the left (a) and the spatiotemporal 2D map is shown to the right (b).

Figures 10 and 11 present another pathological case, coronary case 2 (CAD2). In this case, it can be noticed that the 3D curves in Figure 10 are changing much slower with time when compared with the two cases presented previously. The ECG signal presented in Figure 11(a) shows three ECG cycles, and the corresponding image in Figure 11(b) contains two brighter regions on the right side of the image. These two regions, each of which is located within one ECG cycle (the 2nd and the 3rd cycles), represent considerably less prominent patterns (faded patterns) than those observed in the previous cases. Especially within the 1st ECG cycle, the corresponding pattern is much more faded than the other two. These observations indicate that the motion of the vessel wall in this case is irregular and much slower than in previous cases.

**Figure 12:**
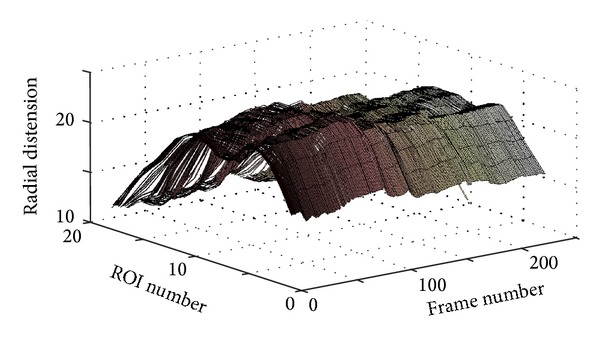
3D plot showing the wave propagation along the vessel wall for the elderly case E1.

**Figure 13:**
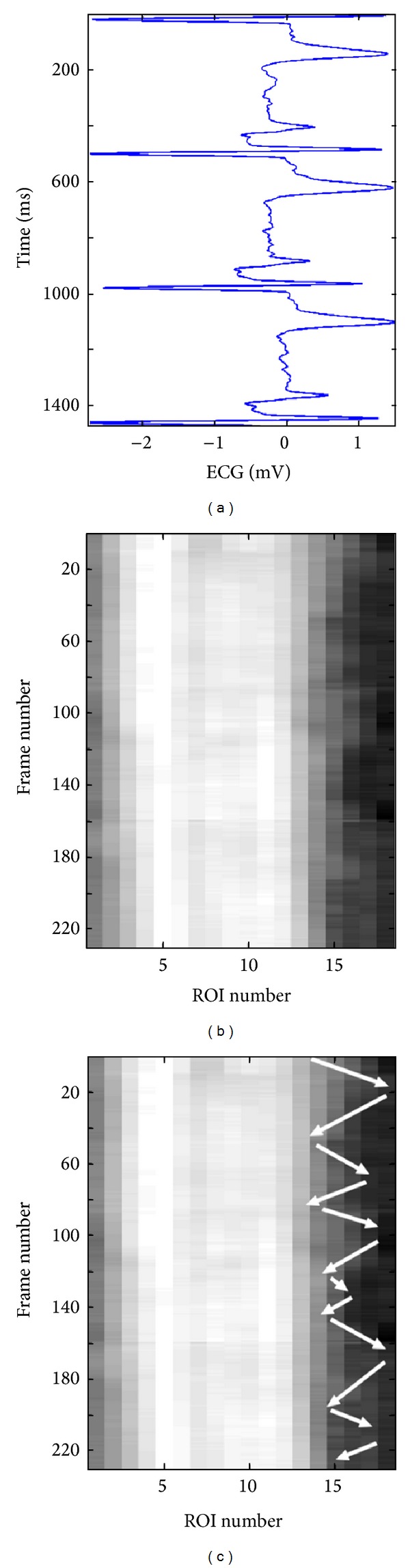
Spatiotemporal wave representation for the elderly case E1 in Figure 12. The ECG signal is shown to the left (a) and the spatiotemporal 2D map is shown to the right (b). The longitudinal motion, across neighboring ROIs, is marked by white arrows on the spatiotemporal 2D map (c).

Finally, Figures 12 and 13 present a healthy elderly case (E1). Here, it can be noted that the 3D curves in Figure 12 are changing even much slower with time compared to those of the coronary case CAD2, presented in Figure 10. Furthermore, the pattern presented in Figure 13(b) is much more faded than the pattern of coronary case (2) presented in Figure 11(b). Therefore, it is not easy to visually recognize the pattern. However, an attempt to mark the pattern (manually with white arrows) is presented in Figure 13(c). This figure shows that the vessel wall seems to perform a right-left motion twice within each ECG cycle. This motion is somewhat regular, but the variation in the corresponding 2D pattern is much slower, much weaker, and clearly different from the triple motion pattern of the healthy young case Y1.

### 2.5. Spatiospectral Wave Representation

This task is based on the transformation of the spatiotemporal wave representation into the frequency domain. The basic idea and purpose of this task is to extract and evaluate some features or parameters that can serve as measures for the characteristics and properties of the mechanical wave signals propagated along the blood vessel wall. This wavy signal or pattern is generated due to changes in blood flow and pressure inside the vessel while pumping blood by the heart.

An efficient way to understand and analyze a signal is by looking at the magnitude of its Fourier spectrum, because it can show if this signal is periodic or irregular, if it is weak or strong (depicting small or large variations), and if it is changing quickly or slowly (i.e., the frequency components obtained). Another important property of the Fourier spectrum is that it is phase-shift invariant, which makes aligning and comparing the properties of a set of signals an easy task.

**Figure 14:**
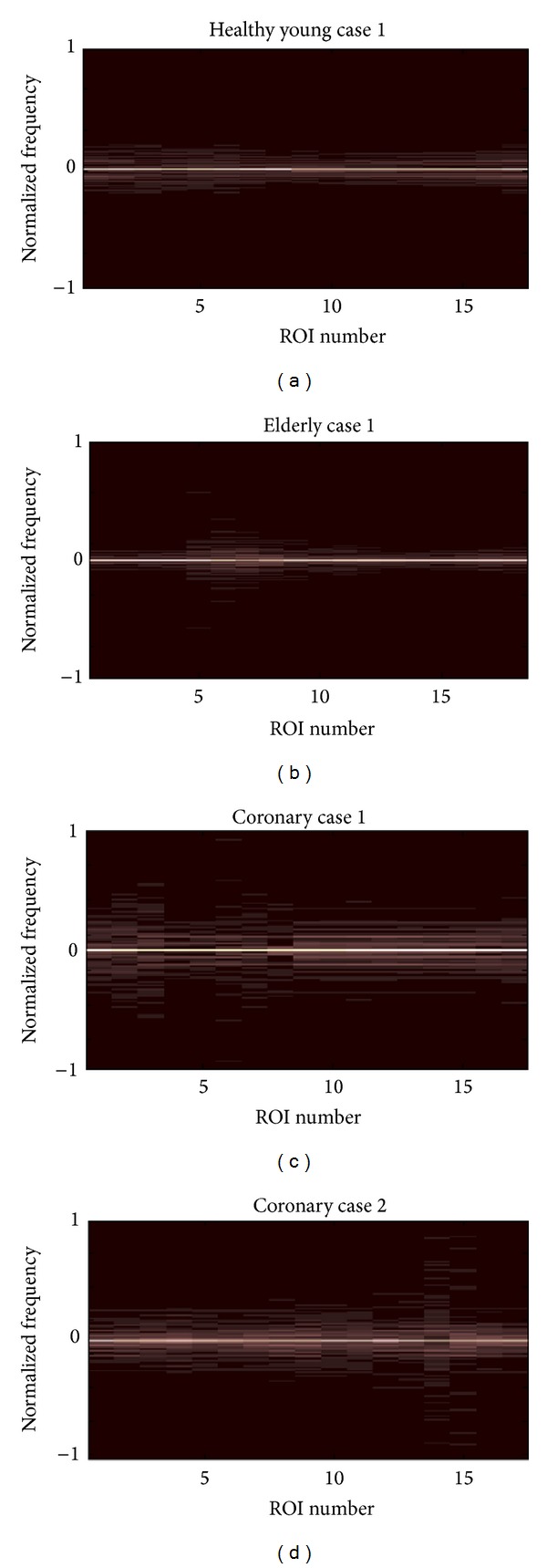
Spatiospectral 2D maps generated when applying the Fourier transform to each column (which corresponds to one ROI) of the spatiotemporal 2D maps shown in Figures 7, 9, 11, and 13.

Figure 14 presents four spatiospectral 2D maps which correspond to the four cases discussed previously in this section as examples representing the three categories considered in this study. Each of these maps is generated by applying the Fourier transform to each column in the corresponding spatiotemporal 2D map. As a result, each radial-variation time-signal of an ROI is replaced by the magnitude of its Fourier spectrum. As an example, Figure 5 shows the chosen ROIs along the wall of the carotid artery for one case. What can be easily observed from this kind of spatiospectral 2D map is whether all ROIs' local radial-motion signals have the same spectral properties or not.

In case all ROIs along a vessel wall have similar or almost similar Fourier spectra, this indicates homogeneous mechanical properties and physiological quality along the vessel wall, which in turn can indicate a healthy status. In Figure 14(a), which shows the spatiospectral 2D map of the healthy young case Y1, it can be observed that all ROIs have almost the same frequency bandwidth and spectral pattern, indicating homogeneous spectral characteristics and mechanical properties along the vessel wall. However, that is not the case in Figures 14(b), 14(c), and 14(d), which show the spatiospectral 2D maps of the healthy elderly case (E1), the pathological elderly case CAD1 (coronary case 1), and the pathological elderly case CAD2 (coronary case 2), respectively. In all these three cases, it is easy to observe nonhomogeneous spectral properties along the vessel walls. The frequency bandwidth is much narrower at a majority of the ROIs in the healthy elderly case E1 (i.e., the motion of these ROIs is slower and smoother), while several ROIs have much wider frequency bandwidths than the healthy young case Y1 (i.e., the motion of these ROIs is faster and not smooth). In the coronary cases, CAD1 and CAD2, the frequency bandwidth is much wider than the healthy young case Y1 at all ROIs. Several ROIs cover almost the whole frequency bandwidth of the Fourier spectra, as in the case of ROI number 6 in CAD1 (Figure 14(c)) and ROIs numbers 14 and 15 in CAD2 (Figure 14(d)).

However, it is possible to automatically evaluate the homogeneity of the spectral characteristics and properties of the spatiospectral 2D maps instead of using visual inspection. By this way, a more objective, accurate, and fast assessment can be achieved. Two straightforward statistical measures can be used for this purpose: a type of mean-value measure in addition to a variance measure across all ROIs (along one blood vessel wall) considered and used for the assessment task.

**Figure 15:**
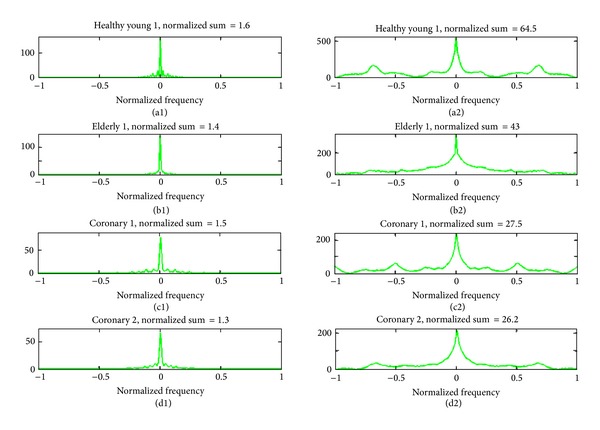
The plots in the left column (a1, b1, c1, and d1) show four spectral curves, each of which is the average of the columns of the spatiospectral 2D map corresponding to one of the four cases presented in Figure 14. The right column shows four plots (a2, b2, c2, and d2), denoted as FT^2^ curves, each of which presents the result of applying the Fourier transform to the corresponding spectral curve in the left column. Normalized sums of the areas under each spectral curve and each FT^2^ curve are also presented.

Since the spectrum of the wavy motion at each ROI is presented as a column in the corresponding spatiospectral 2D map, the average of all columns (i.e., average of spectra for all ROIs) of the map is calculated and analyzed. In Figure 15, the plots in the left column (a1, b1, c1, and d1) show four spectral curves, each of which is the average of the columns of the spatiospectral 2D map of one of the four cases presented in Figure 14. The normalized value of the sum of the areas under each resulting spectral curve (i.e., for each case) is also calculated and presented. But, inspecting these values shows mainly the difference between young and elderly cases. In other words, these results show that using only the sums under these curves is not enough to differentiate between the three categories considered in the current study. Therefore, more advanced analysis methods should be used to achieve this purpose.

An efficient approach to reveal the characteristics of the shape of a curve is through extracting its spectral properties by utilizing the Fourier transform (FT) and computing its spectrum. Some results of applying this approach are presented in the right column of Figure 15 where four plots are shown (a2, b2, c2, and d2), denoted as FT^2^ curves, because these results are obtained by applying FT twice on the data (i.e., FT^2^ means computing the FT of the average of FTs). Each one of these four plots (shown in Figures 15(a2), 15(b2), 15(c2), and 15(d2)) presents the result of applying FT to the corresponding spectral curve in the left column of the same figure (Figure 15). Normalized values of the sums of the areas under each resulting FT^2^ curve are also presented in each subfigure, showing noticeable differences between the three categories of cases: the healthy young case Y1, the healthy elderly case E1, and the two coronary cases, CAD1 and CAD2.

**Figure 16:**
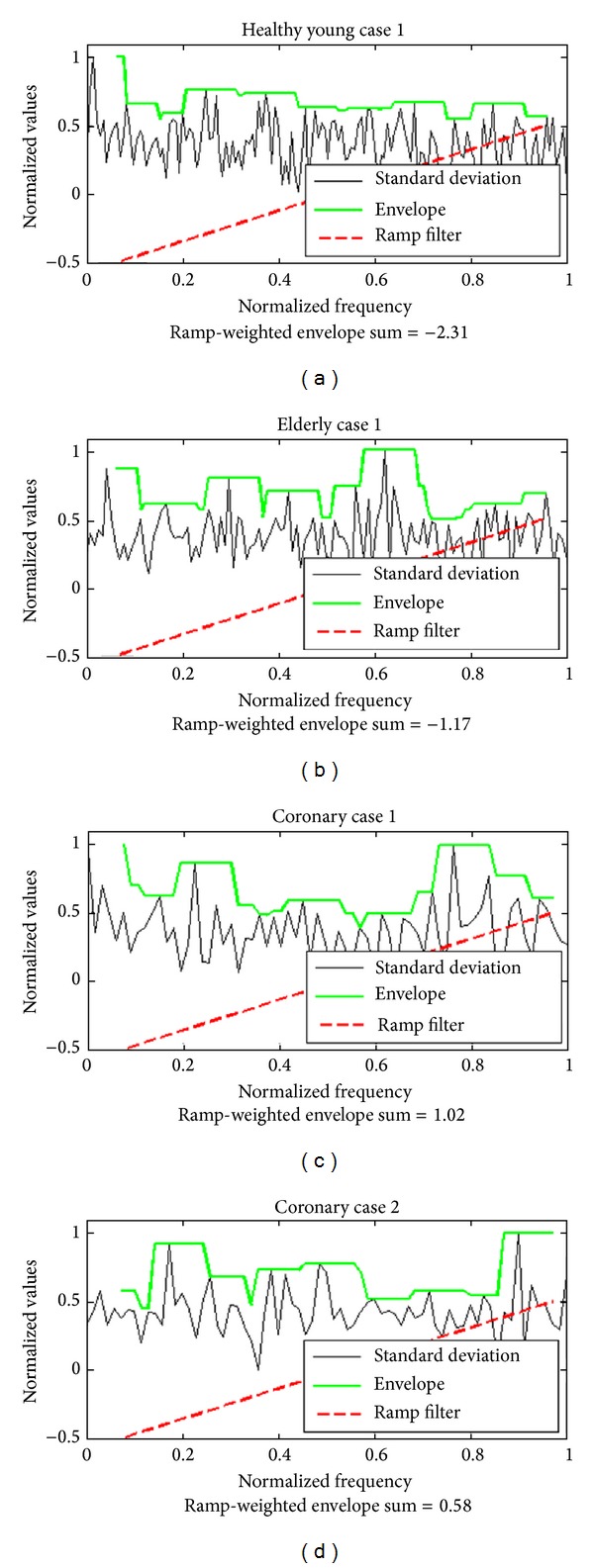
The plots show the standard deviation curves and the corresponding envelope curves for the cases presented in Figure 15. For each plot, the normalized sum of the areas under the envelope curve weighted by a ramp filter is also presented.

For the variance measure, the standard deviation of all columns of the 2D map (i.e., the standard deviation of the spectra for all ROIs) is calculated and analyzed. Therefore, each value of the resulting vector is the standard deviation of one row in the 2D map. In order to simplify the analysis to measure the overall form of a standard deviation curve, it can be normalized and its envelope curve can be computed. Furthermore, higher frequencies can be given more importance than the lower ones when evaluating the nonhomogeneity of the spectral properties along the blood vessel wall. As a classification feature, ramp-weighted normalized sums of the areas under the envelope curve of the standard deviation are computed for all cases.  Figure 16 shows four plots that correspond to the four cases presented in Figure 14. The values of this new feature are obtained and presented in Figure 16, showing that this feature can be used as an efficient classification parameter to differentiate between the three categories considered in the current study.

## 3. Results

In this section, the methods proposed, discussed, and tested in the previous sections are utilized to classify the three types of cases or categories that are available for the current study. Totally, 30 healthy young cases, 47 healthy elderly cases, and 69 pathological elderly cases (suffering from coronary artery disease) were analyzed and classified using these methods.

**Figure 17:**
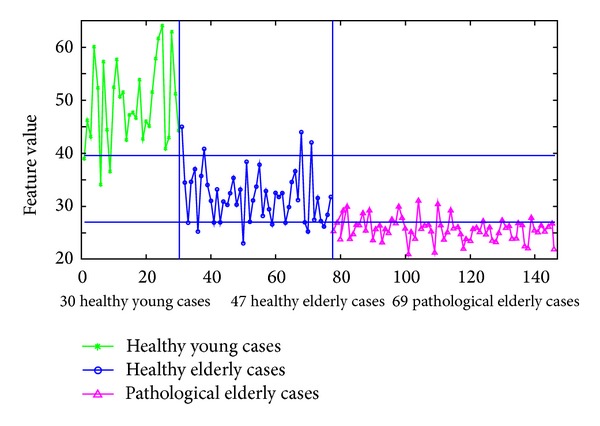
Differentiation between healthy young, healthy elderly, and pathological elderly cases (suffering from CAD), when using the normalized sum of the areas under the corresponding FT^2^ curve for each case, as a classification feature or parameter.

**Figure 18:**
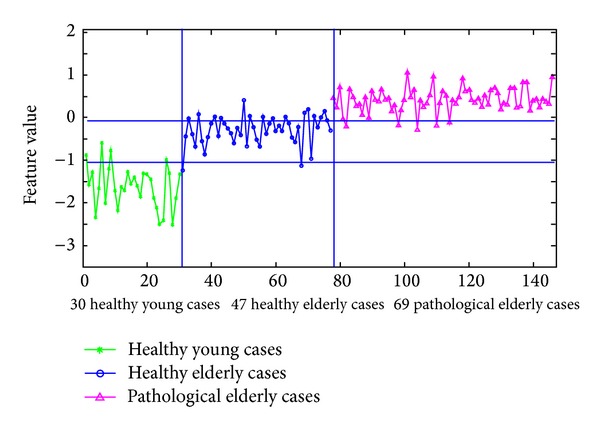
Differentiation between healthy young, healthy elderly, and pathological elderly cases (suffering from CAD), when using the normalized sum of the areas under the corresponding standard deviation curve's envelope for each case, as a classification feature or parameter.

Figures 17 and 18 summarize the performance of using the normalized sums of the areas under the corresponding FT^2^ curve and the standard deviation curve's envelope for each case as a classification feature or parameter to differentiate between these three categories of cases. In both figures, it is much easier to differentiate the healthy young cases from the elderly ones than differentiating the pathological elderly cases from the healthy elderly ones. Therefore, the following analysis will focus on differentiating between pathological and healthy elderly cases.

In Figure 17, the feature values are between 21.5 and 32.1 for the pathological elderly cases and between 22.7 and 45 for the healthy elderly cases. When considering the lower horizontal separation line (at a feature value of 27.3) in Figure 17 as a threshold to identify pathological elderly cases among all elderly cases in the current study, a sensitivity of 78.3% and a specificity of 74.5% can be obtained. On the other hand, for the 30 healthy young cases, the corresponding feature values are between 33.8 and 64. This shows that the healthy young cases can be easily separated from the pathological elderly cases with 100% accuracy. Furthermore, the overlap extent, between the two sets of feature values for the healthy young cases and the healthy elderly cases, is much less than the corresponding overlap between healthy and pathological elderly cases.

In the same way, in Figure 18, the feature values for the pathological elderly cases and for the healthy elderly cases are between −0.22 and 1.19 and between −1.24 and 0.4, respectively. When considering the higher horizontal separation line (at a feature value of −0.1) in Figure 18 as a threshold to identify pathological elderly cases among all elderly cases, a sensitivity of 92.8% and a specificity of 68.1% can be obtained. Furthermore, the feature values are below −0.5 for all healthy young cases, showing that these cases can be separated with 100% accuracy from the pathological elderly cases. In addition, the overlap between the two sets of feature values of the young and the elderly healthy cases is much less than the overlap between the healthy and the pathological elderly cases.

However, to achieve better results, it is possible to combine the results in both Figures 17 and 18 so that it is enough for a case to be true positive or true negative in one of these two figures. In other words, by considering the resulting feature values and the chosen classification thresholds presented in these figures, a sensitivity of 92.8% and a specificity of 76.6% can be obtained.

The pairwise Student's *t*-test and the pairwise ANOVA test were performed, at each time, using two sets of 47 pairs of values (for pathological elderly cases and healthy elderly cases) for each of the two features discussed and used previously in this work. That is, pairs of the feature values of all 47 healthy elderly cases and a randomly chosen set of 47 feature values of pathological elderly cases were used in these tests. These two statistical analysis tests resulted in *P* values much less than 0.0001 indicating a significant difference between each of such two sets of 47 feature values.

**Figure 19:**
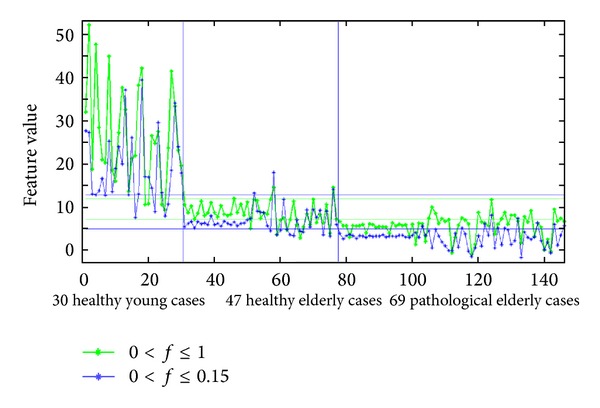
Differentiation between healthy young cases, healthy elderly cases, and pathological elderly cases (suffering from CAD), when using the two spectral area measures for normalized frequencies 0 < *f* ≤ 1 (green curve) and 0 < *f* ≤ 0.15 (blue curve) as proposed in [31].

In addition, for comparison, the method proposed in [31] was used to differentiate between the three classes of cases in all datasets available for this work. The results of using the two spectral area measures, proposed in [31], for normalized frequencies 0 < *f* ≤ 1 (green curve) and 0 < *f* ≤ 0.15 (blue curve) are presented in Figure 19. The thresholds chosen and used for classification using these two spectral area measures are shown as two green horizontal lines for the approach considering 0 < *f* ≤ 1 as two blue horizontal lines for 0 < *f* ≤ 0.15. The corresponding sensitivity and specificity measures for this method were 78.3% and 74.5%, respectively, for 0 < *f* ≤ 1, while these classification quality measures were 85.5% and 78.7%, respectively, for 0 < *f* ≤ 0.15. What these results reveal is that the method proposed in [31] is optimized for the smaller dataset used in that study. Therefore, the results obtained in that study were optimal achieving 100% accuracy, compared to 82.8% when applying the approach considering the normalized frequencies 0 < *f* ≤ 0.15 in the current work.

## 4. Conclusions

Two approaches for visualizing the wave patterns temporally and spatially, as well as spectrally and spatially along the walls of the carotid artery, were presented. A systematic procedure was utilized to divide the vessel wall longitudinally into small regions of the same size and overlapping midway. Each of these regions was evaluated in terms of radial variation (radial distension). Two means of visualizing these patterns were presented, namely, a 2D spatiotemporal wave-representation map and a 2D spatiospectral wave-representation map.

An automated procedure was developed to provide a new method of characterizing the wavy motion of the carotid artery wall. The method involved capturing the progression of the wave patterns that appear both temporally and spatially along the vessel wall by measuring radial distension, at each time point (i.e., in each frame of the ultrasonic video), within each one of the overlapping small regions. By this way, a 2D spatiotemporal map, using pseudo colors, was generated for the case at hand, which belongs to one of the three categories of cases considered in this study. The spectral characteristics of these wavy patterns, which are related to the physiological properties along the carotid artery wall, were obtained by using the Fourier transform to compute the spectrum of the radial-variation signal of each small region. The result is a pseudocolored 2D spatiospectral map for the corresponding case. The resulting 2D maps of these two automated visualization methods can be used to aid in the noninvasive diagnosis and detection of pathological conditions in the cardiovascular system in two ways. Firstly, for each subject, visual inspection of the corresponding two 2D maps can help the cardiologist understand the physiological condition of the examined arterial wall. In addition, it is also possible to utilize an automated method to compute a number of useful features that can be used to evaluate and classify the physiological condition of the arterial wall.

The findings obtained in this study show that there are inherent differences in the way a wave progresses along an arterial vessel wall between healthy and pathological cases. It is evident that, in pathological elderly cases, the wave does not propagate as it would be normally expected as in healthy cases; for example, it is often skewed and/or damped due to plaques, impediments, and/or stiffness of the artery.

The differences that can be observed in the resulting 2D patterns indicate that such spatiotemporal and spatiospectral representations can be utilized to simplify the task of medical specialists in detecting any defects in the condition of the carotid artery through visualizing, inspecting, interpreting, analyzing, and comparing these patterns with those of normal healthy cases. This automated method, which is based on 2D spatiotemporal and spatiospectral visualization, can also be used to assess the progression of a given treatment by inspecting and comparing the corresponding 2D maps of the same case before and after the treatment.

The proposed method for automated evaluation of the homogeneity and the variation in the patterns of the spatiospectral maps resulted in a sensitivity of 97.1% and a specificity of 74.5%, compared to 85.5% and 78.7%, respectively, when employing the method proposed in [31]. The specificity of the method presented was moderate because it was based on the general basic principle of studying the characteristics of the wave propagation along the carotid artery. The hypothesis that was suggested and tested in this work was that, in subjects suffering from CAD, abnormal physiological function of the cardiovascular system would cause disturbances to the physical properties of the wave propagation process through arterial vessel walls in general and along the carotid artery in particular.

The subjects that were considered included CAD cases as identified by pathologists. Pathological wave propagation may give indication to CAD and serious vascular problems, and, in this paper, it has been demonstrated that the type of abnormal or disturbed wave propagation in healthy elderly subjects is significantly different than that in elderly subjects with CAD.

However, the sensitivity and specificity of this novel automated method can be enhanced by defining a new category of suspected cases, with feature values in between those of the pathological elderly and the healthy elderly cases. The reliability of this method is confirmed by the fact that all healthy young cases can be separated from the pathological cases with an accuracy of 100%.

The advantage of this approach of presenting the 2D patterns of the wavy motion of the arterial vessel wall is that it can provide much more information (temporally, spectrally, and spatially) than the wave intensity signal concerning the progression of the wave and the spatially localized condition of the wall of the carotid artery. However, accurate physiological insight is required to be able to interpret such 2D maps and draw deductions from these types of spatiotemporal and spatiospectral visualization approaches.

The automation of the feature extraction process in addition to the automatic classification to differentiate between healthy and pathological cases would significantly simplify the task of medical specialists in identifying abnormal motion of arterial vessel walls and detecting cardiovascular symptoms. This approach has a number of desired properties, such as being noninvasive, fully automated, fast, user friendly, intuitive, straightforward, robust, reproducible, and objective.

Finally, this approach can be developed further to build a computer-aided decision support system for the automatic classification and diagnosis of different arterial characteristics and physiological conditions among different categories of patients (e.g., to target even narrower categorization or early stages of atherosclerosis) with high accuracy.
