# Corrigendum to “Automatic Characterization of the Physiological Condition of the Carotid Artery in 2D Ultrasound Image Sequences Using Spatiotemporal and Spatiospectral 2D Maps”

**DOI:** 10.1155/2017/4237858

**Published:** 2017-06-28

**Authors:** Hamed Hamid Muhammed, Jimmy C. Azar

**Affiliations:** ^1^School of Technology and Health (STH), Royal Institute of Technology (KTH), Alfred Nobels Alle 10, SE-141 52 Huddinge, Sweden; ^2^Centre for Image Analysis, Uppsala University, P.O. Box 337, 751 05 Uppsala, Sweden

In the article titled “Automatic Characterization of the Physiological Condition of the Carotid Artery in 2D Ultrasound Image Sequences Using Spatiotemporal and Spatiospectral 2D Maps” [1], there were errors in the sensitivity and specificity values and missing information on how they were derived from Figures 17, 18, and 19.

Information on how these values were obtained is explained as follows:

  True positive (TP) is the number of sick individuals who are correctly identified as sick.
  False positive (FP) is the number of healthy individuals incorrectly identified as sick.
  True negative (TN) is the number of healthy individuals correctly identified as healthy.
  False negative (FN) is the number of sick individuals incorrectly identified as healthy.
  Sensitivity = TP/(TP + FN) = TP/(total number of sick individuals).
  Specificity = TN/(TN + FP) = TN/(total number of healthy individuals).

The thresholds (the separation lines) in Figures 17 and 18 are calculated as the mean value of all sick and healthy individuals in the study. From Figure 17, the following values are obtained:

  FN = 15
  TP = 69 − 15 = 54
  FP = 12
  TN = 47 − 12 = 35

Then

  Sensitivity = TP/69 = 0.7826
  Specificity = TN/47 = 0.7447

From Figure 18, the following values are obtained:

  FN = 5
  TP = 69 − 5 = 64
  FP = 15
  TN = 47 − 15 = 32

Then

  Sensitivity = TP/69 = 0.9275
  Specificity = TN/47 = 0.6809

And when combining Figures 17 and 18, the following values are obtained:

  FN = 5
  TP = 69 − 5 = 64
  FP = 12
  TN = 47 − 12 = 35

Then

  Sensitivity = TP/69 = 0.9275
  Specificity = TN/47 = 0.7447

This combining rule is equivalent to a logical “OR.” In Figures 17 and 18, there are 5 out of 69 pathological elderly cases that are misclassified simultaneously in both these figures whereas the remaining 64 cases appear correctly classified in at least one of the two figures. This implies that FN = 5 and TP = 69 − 5 = 64, so the combined sensitivity is equal to (64/69)*∗*100 = 92.8%. On the other hand, there are 12 out of 47 healthy elderly patients who happen to be misclassified simultaneously in both these figures, which implies that FP = 12 and TN = 47 − 12 = 35, so the combined specificity is equal to (35/47)*∗*100 = 74.5%.

From the green curve in Figure 19, the following values are obtained:

  FN = 15
  TP = 69 − 15 = 54
  FP = 12
  TN = 47 − 12 = 35

Then

  Sensitivity = TP/69 = 0.783
  Specificity = TN/47 = 0.745

From the blue curve in Figure 19, the following (better) values are obtained:

  FN = 10
  TP = 69 − 10 = 59
  FP = 10
  TN = 47 − 10 = 37

Then

  Sensitivity = TP/69 = 0.855
  Specificity = TN/47 = 0.787

Therefore, the sensitivity and specificity values should be corrected in the following sections:

- In* Abstract*, the sensitivity value in the sentence “Automatic differentiation, between cases of these three categories, was achieved with a sensitivity of** 97.1%** and a specificity of 74.5%.” should be corrected to “Automatic differentiation, between cases of these three categories, was achieved with a sensitivity of** 92.8%** and a specificity of 74.5%.”
- In the fifth paragraph of* Results*, the specificity value in the sentence “In other words, by considering the resulting feature values and the chosen classification thresholds presented in these figures, a sensitivity of 92.8% and a specificity of** 76.6%** can be obtained.” should be corrected to “In other words, by considering the resulting feature values and the chosen classification thresholds presented in these figures, a sensitivity of 92.8% and a specificity of** 74.5%** can be obtained.”
- In the last paragraph of* Results*, the sensitivity value in the sentence “Therefore, the results obtained in that study were optimal achieving 100% accuracy, compared to** 82.8%** when applying the approach considering the normalized frequencies 0 < *f* ≤ 0.15 in the current work.” should be corrected to “Therefore, the results obtained in that study were optimal achieving 100% accuracy, compared to** 85.5%** when applying the approach considering the normalized frequencies 0 < *f* ≤ 0.15 in the current work.”
- In the fifth paragraph of* Conclusions*, the sensitivity value in the sentence “The proposed method for automated evaluation of the homogeneity and the variation in the patterns of the spatiospectral maps resulted in a sensitivity of** 97.1%** and a specificity of 74.5%, compared to 85.5% and 78.7%, respectively, when employing the method proposed in [31].” should be corrected to “The proposed method for automated evaluation of the homogeneity and the variation in the patterns of the spatiospectral maps resulted in a sensitivity of** 92.8%** and a specificity of 74.5%, compared to 85.5% and 78.7%, respectively, when employing the method proposed in [31].”
